# Optimization of Process Parameters of Extraction of Amentoflavone, Quercetin and Ginkgetin from *Taxus chinensis* Using Supercritical CO_2_ Plus Co-Solvent

**DOI:** 10.3390/molecules191117682

**Published:** 2014-10-31

**Authors:** Xiao Ruan, Liu-Ye Yan, Xian-Xian Li, Ben Liu, Huan Zhang, Qiang Wang

**Affiliations:** Ningbo Institute of Technology, Zhejiang University, Ningbo, 315100, China; E-Mails: ruanxiao@nit.zju.edu.cn (X.R.); yanliuye@163.com (L.-Y.Y.); lixianxian@163.com (X.-X.L.); liuben@nit.zju.edu.cn (B.L.); zhhuan@nit.zju.edu.cn (H.Z.)

**Keywords:** supercritical fluid extraction, central composite design, flavonoids, *Taxus chinensis*

## Abstract

The effects of extraction time, temperature, pressure and different concentration of ethanol and their interactions on the yields of amentoflavone, quercetin and ginkgetin extracted from *Taxus chinensis* by supercritical CO_2_ were investigated by using a central composite design (CCD). An CCD experimental design with four factors and five levels was used to optimize the extraction parameters. Ultra performance liquid chromatography (UPLC) was used to analyze the content of the tree components in the extracts. Experimental results show that the main effects of factors and their interactions are significant on the yields (*p* < 0.05). The optimal extraction conditions were established for the three compounds: yield of 4.47 mg/g for amentoflavone at 48 °C, 25 MPa, 2.02 h and 78.5% ethanol, 3.73 mg/g for quercetin at 46 °C, 24 MPa, 2.3 h, 82% ethanol and 3.47 mg/g for ginkgetin at 48 °C, 20 MPa, 2.38 h, 82% ethanol, respectively.

## 1. Introduction

The flavonoids are one of the largest known groups of natural products, which are widely distributed in various plants [1,2]. One of the main interests in the pharmaceutical industry is that the compounds possess many biological activities such as scavenging of free radicals, immune modulation and hormone action and that they can serve as a starting point for the development of optimal derivatives [3,4,5,6]. *Taxus* is an important natural resource for the extraction of taxoids, which have been utilized as anticancer agents [7]. The increasing need of taxoids has leds to increasing amount of the extracts free of taxoids from *Taxus* [8]. It was later found that high contents of flavonoids exist in *Taxus*, which is significant to extract the flavonoids for food and pharmaceutical applications [9].

Supercritical CO_2_ extraction is currently regarded as a natural and green technique for natural product extraction, and an important alternative to conventional separation methods, not only because it is simpler, faster, and more efficient, but also because it does not require the consumption of large amounts of organic solvents which are both expensive and potentially harmful. The technique has been applied to extract bioactive compounds from natural resources [10].

**Figure 1:**
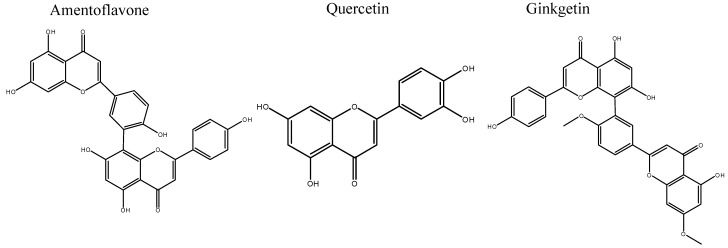
Structures of Amentoflavone, Quercetin and Ginkgetin.

The aim of this study is to investigate the effects of extraction time, temperature, pressure and different concentration of ethanol in supercritical CO_2_ extraction on the yields of amentoflavone, quercetin and ginkgetin (Figure 1) by applying a central composite design method. The method is a collection of statistical and mathematical methods that are useful for developing, improving and optimizing a process. Its main advantage is the reduced number of experimental trials required to assess multiple parameters and their interactions [11,12]. To our best knowledge, Taxus flavonoids from *Taxus chinensis* were extracted by SFE and analyzed with UPLC for the first time in this study.

## 2. Results and Discussion

### 2.1. UPLC Chromatogram

**Figure 2:**
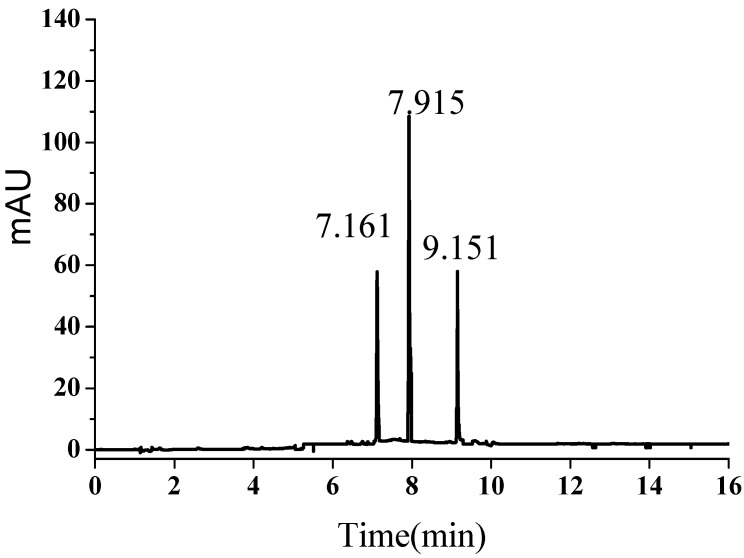
Ultra performance liquid chromatogram of extract obtained with CO_2_ plus ethanol. 1. Quercetin; 2. Amentoflavone and 3. Ginkgetin.

The UPLC profile of a SFE extract of the powdered leaves of *T. chinensis* is seen in Figure 2. Based on the available standards of quercetin, amentoflavone and ginkgetin, it is possible to identify the corresponding peaks, which appear at retention times of approximately 7.16 min, 7.92 min and 9.15 min, respectively. The extracts obtained by using SFE under different conditions show chromatograms similar to Figure 2.

### 2.2. Selection of Extraction Time

**Figure 3:**
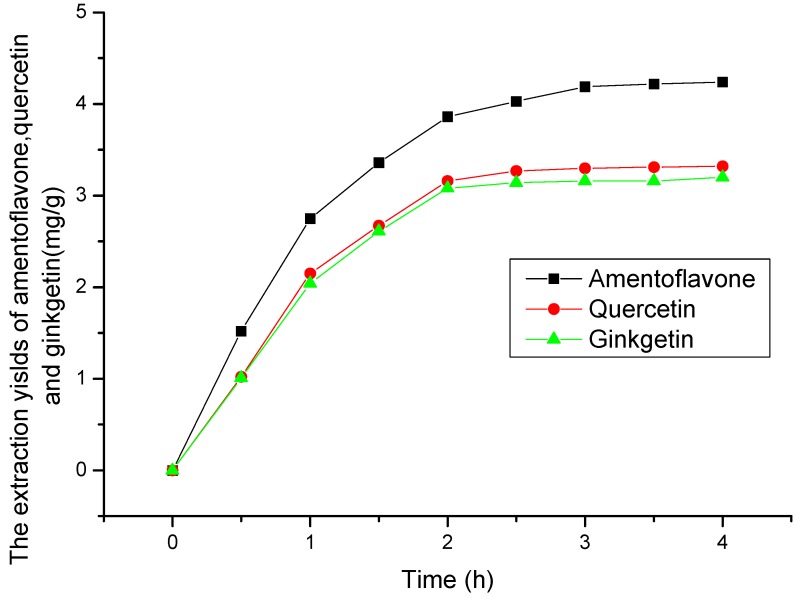
The effect of extraction time on the yields of amentoflavone, quercetin and ginkgetin at temperature 50 °C, pressure 20 MPa and CO_2_ + 75% ethanol.

Figure 3 displays the effect of extraction time on the yields of amentoflavone, quercetin and ginkgetin. It is possible to extract >95% of them within 3 h (all yields being based on the recovery obtained after 4 h), therefore an extraction time <3 h was selected in this study. It can be observed that fast extraction of the flavonoids occurs within 2 h.

### 2.3. Model Fitting and Evaluation of the Factors

The main objective of this study was to investigate the process parameters for maximum extraction of amentoflavone, quercetin and ginkgetin from *T. chinensis* using SFE. The experimental design and values of independent variables are shown in Table 1. As analyzed using ANOVA (Table 2), the models were highly significant with very low *p*-values (*p* < 0.0001) with R^2^ value 0.9892, 0.9832, and 0.9849, respectively, for the extraction of the components. These values indicated that the polynomial regression models were suitable to determine optimum conditions for the extraction of the three components. As seen from Table 2, the *p*-values of the four main variables are less than 0.05. Therefore, extraction time, temperature, pressure and ethanol concentration have significant effects on the yields of the three components. Interactions of time × pressure (X_1_X_3_) and pressure × ethanol concentration (X_3_X_4_) are significant (*p* < 0.05) while the other interactions are insignificant in the model for the extraction of amentoflavone. Interaction of pressure × ethanol concentration (X_3_X_4_) are significant for the extraction of quercetin. Interactions of time × ethanol concentration (X_1_X_4_), temperature × pressure (X_2_X_3_) and temperature × ethanol concentration (X_2_X_4_) are significant for the extraction of ginkgetin.

Fitting a regression surface to the experimental results, the following Equations (1)–(3) were obtained, applicable to predict the achievable amentoflavone yield (*Y_1_*), quercetin yield (*Y_2_*) and ginkgetin yield (*Y_3_*), respectively as a function of the studied process variables:

*Y_1_* (mg/g) = 4.34 + 0.24X_1_ + 0.09X_2_ + 0.26X_3_ + 0.04X_4_ − 0.14X_1_^2^ − 0.058X_2_^2^ − 0.14X_3_^2^ − 0.34X_4_^2^ − 5.63 × 10^−^3 X1X2 − 0.043X_1_X_3_ − 0.021X_1_X_4_ + 0.033X_2_X_3_ − 0.017 X_2_X_4_ − 0.047X_3_X_4_ (1)

*Y_2_* (mg/g) = 3.54 + 0.16X_1_ + 0.094X_2_ + 0.19X_3_ − 0.13X_4_ − 0.059X_1_^2^ − 0.035X_2_^2^ − 0.079X_3_^2^ − 0.49X_4_^2^ + 0.018X_1_X_2_ + 0.042X_1_X_3_ + 6.25×10^−4^ X1X4 − 0.046X_2_X_3_ + 0.051X_2_X_4_ + 0.18X_3_X_4_ (2)

*Y_3_* (mg/g) = 3.27 + 0.13X_1_ + 0.16X_2_ + 0.15X_3_ + 0.083X_4_ − 0.076X_1_^2^ − 0.12X_2_^2^ − 0.12X_3_^2^ − 0.55X_4_^2^ + 0.048X_1_X_2_ + 0.016X_1_X_3_ + 0.078X_1_X_4_ + 0.097X_2_X_3_ − 0.061X_2_X_4_ − 0.066X_3_X_4_ (3)

A positive sign indicates a synergistic effect while negative sign represents an antagonistic effect of the factor on the response of the model. Therefore, the main effects of extraction time, temperature, pressure and ethanol concentration have positive effects on amentoflavone and ginkgetin yield. Extraction time, temperature and pressure have positive effects, while the main effect of ethanol concentration plays a negative role on quercetin yield.

**Table 1:** Experimental design using central composite design with corresponding experimental results.

Trail No.	X_1_	X_2_	X_3_	X_4_	Amentoflavone	Quercetin	Ginkgetin
	Time (h)	Temperature (°C)	Pressure (bar)	Ethanol Concentration (ethanol/water, v/v, %)	Yield (mg/g)	Yield (mg/g)	Yield (mg/g)
1	2	45	20	100	3	1.52	1.19
2	2	45	20	60	3.01	1.64	1.11
3	2	45	20	80	4.35	3.53	3.27
4	2	45	30	80	4.37	3.56	3.21
5	2.5	40	25	70	3.92	3.28	2.1
6	1.5	50	15	90	3.29	2.32	2.19
7	1	45	20	80	3.28	2.91	2.74
8	2	35	20	80	3.95	3.21	2.57
9	1.5	50	25	70	3.83	2.84	2.61
10	1.5	40	25	90	3.62	2.82	2.18
11	2.5	50	25	70	4.22	3.31	2.97
12	2.5	40	25	90	4.02	3.13	2.55
13	2	45	20	80	4.35	3.53	3.27
14	2	55	20	80	4.35	3.57	3.18
15	2.5	40	15	70	3.55	3.02	1.89
16	1.5	40	25	70	3.51	2.87	2.16
17	1.5	40	15	70	2.93	2.89	1.96
18	1.5	40	15	90	3.23	2.02	2.22
19	1.5	50	25	90	3.84	3.01	2.54
20	2	45	20	80	4.35	3.54	3.26
21	2	45	20	80	4.34	3.53	3.27
22	2.5	50	15	70	3.62	3.22	2.22
23	2	45	10	80	3.24	2.87	2.57
24	2.5	50	15	90	3.83	2.68	2.65
25	2	45	20	80	4.33	3.54	3.27
26	1.5	50	15	70	3.05	3.05	2.11
27	2.5	50	25	90	4.12	3.34	2.96
28	3	45	20	80	4.39	3.68	3.35
29	2.5	40	15	90	3.67	2.21	2.56
30	2	45	20	80	4.33	3.54	3.27

**Table 2:** ANOVA analysis of four parameters for SFE.

Source	Amentoflavone				Quercetin				Ginkgetin			
	Sum of Squares	F-value	*p*-value	R^2^	Sum of Squares	F-value	*p*-value	R^2^	Sum of Squares	F-value	*p*-value	R^2^
Model	7.01	98.42	<0.0001	0.9892	9.36	62.56	<0.0001	0.9832	10.51	69.83	<0.0001	0.9849
X_1_	1.44	282.28	<0.0001		0.64	59.6	<0.0001		0.41	38.44	<0.0001	
X_2_	0.19	37.87	<0.0001		0.21	19.74	0.0005		0.62	57.42	<0.0001	
X_3_	1.59	311.87	<0.0001		0.87	81.42	<0.0001		0.53	48.82	<0.0001	
X_4_	0.039	7.71	0.0141		0.42	39.67	<0.0001		0.16	15.34	0.0014	
X_1_X_2_	0.000506	0.1	0.7567		0.0053	0.49	0.4939		0.037	3.44	0.0832	
X_1_X_3_	0.03	5.85	0.0287		0.028	2.62	0.1260		0.0039	0.36	0.5558	
X_1_X_4_	0.0068	1.34	0.2654		6.3E-06	0.00058	0.9810		0.098	9.08	0.0087	
X_2_X_3_	0.018	3.45	0.0829		0.033	3.12	0.0979		0.15	13.96	0.0020	
X_2_X_4_	0.0045	0.9	0.3589		0.041	3.84	0.0690		0.059	5.47	0.0336	
X_3_X_4_	0.035	6.91	0.0190		0.54	50.89	<0.0001		0.069	6.41	0.0230	
X_1_^2^	0.51	100.27	<0.0001		0.096	8.95	0.0091		0.16	14.87	0.0016	
X_2_^2^	0.091	17.89	0.0007		0.034	3.2	0.0938		0.39	36.02	<0.0001	
X_3_^2^	0.57	111.6	<0.0001		0.17	16.04	0.0011		0.36	33.79	<0.0001	
X_4_^2^	3.24	637.63	<0.0001		6.53	610.66	<0.0001		8.3	771.68	<0.0001	

**Figure 4:**
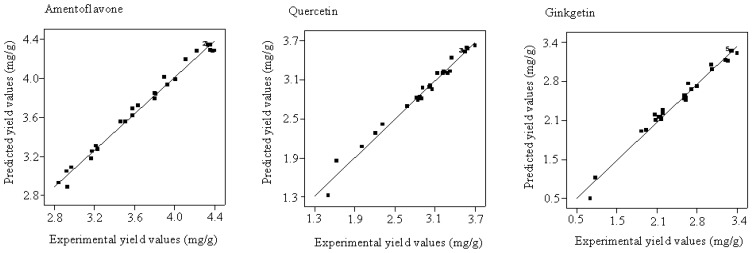
Correlation graph between the predicted and experimental yield values.

A correlation graph shows that a high correlation exists between the experimental and predicted values (see Figure 4). Each point is close to the regression line, which indicates the good fit of the model. Based on Equations (2)–(4), the optimal extraction conditions are 48 °C, 25 MPa, 2.02 h and 78.5% ethanol with the yield of 4.47 mg/g for amentoflavone extraction; 46 °C, 24MPa, 2.3 h, 82% ethanol with the yield of 3.73 mg/g for quercetin and 48 °C, 20MPa, 2.38 h, 82% ethanol with the yield of 3.47 mg/g for ginkgetin.

**Figure 5:**
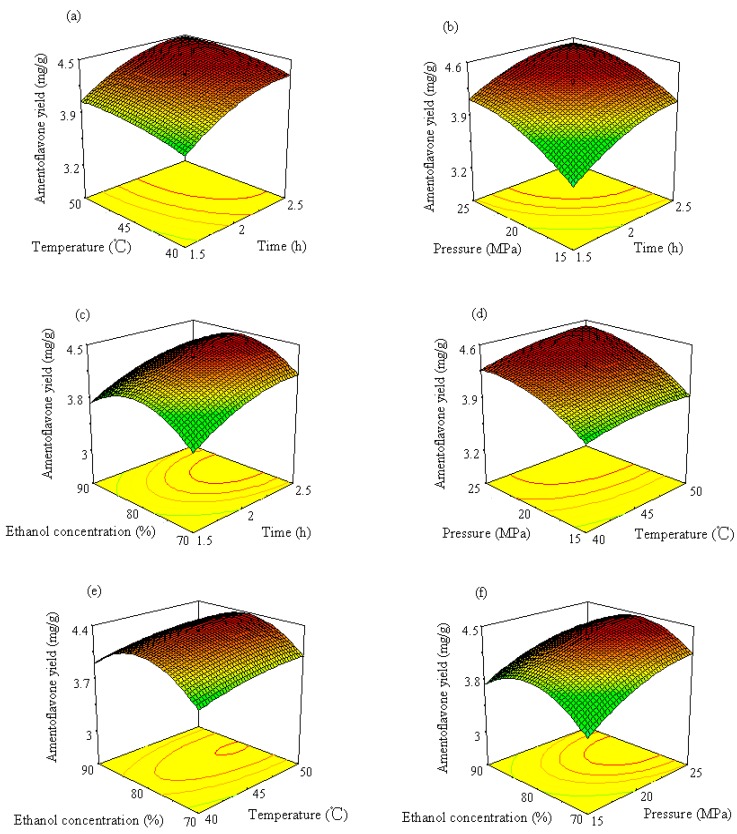
Response surface and contour plots of amentoflavone showing (**a**) the effect of time and temperature at constant 20 MPa and 80% ethanol, (**b**) the effect of time and pressure at constant 45 °C and 80% ethanol, (**c**) the effect of time and ethanol concentration at constant 45 °C and 20 MPa, (**d**) the effect of temperature and pressure at constant 2 h extraction and 80% ethanol, (**e**) the effect of temperature and ethanol concentration at constant 2 h extraction and 20 MPa and (**f**) the effect of pressure and ethanol concentration at constant 2 h extraction and 45 °C.

**Figure 6:**
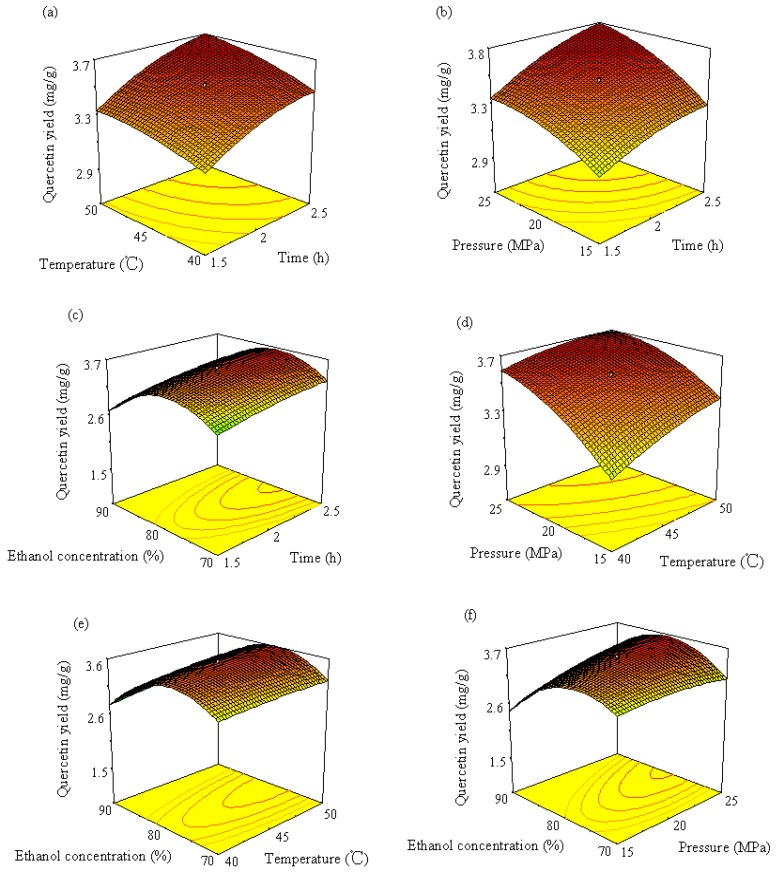
Response surface and contour plots of quercetin showing (**a**) the effect of time and temperature at constant 20 MPa and 80% ethanol, (**b**) the effect of time and pressure at constant 45 °C and 80% ethanol, (**c**) the effect of time and ethanol concentration at constant 45 °C and 20 MPa, (**d**) the effect of temperature and pressure at constant 2 h extraction and 80% ethanol, (**e**) the effect of temperature and ethanol concentration at constant 2 h extraction and 20 MPa and (**f**) the effect of pressure and ethanol concentration at constant 2 h extraction and 45 °C.

**Figure 7:**
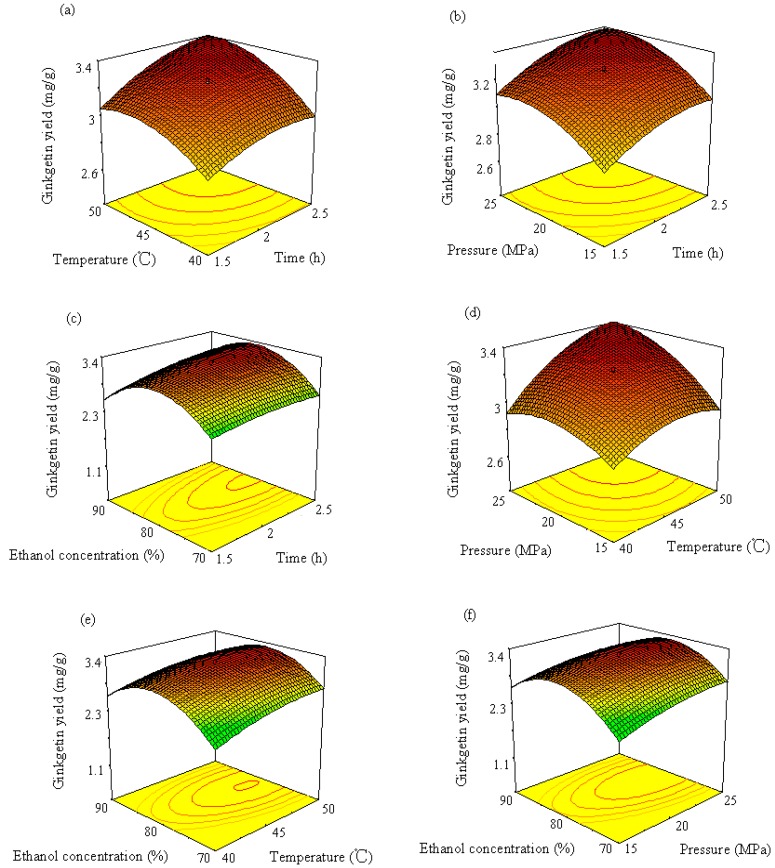
Response surface and contour plots of ginkgetin showing (**a**) the effect of time and temperature at constant 20 MPa and 80% ethanol, (**b**) the effect of time and pressure at constant 45 °C and 80% ethanol, (**c**) the effect of time and ethanol concentration at constant 45 °C and 20 MPa, (**d**) the effect of temperature and pressure at constant 2 h extraction and 80% ethanol, (**e**) the effect of temperature and ethanol concentration at constant 2 h extraction and 20 MPa, and (**f**) the effect of pressure and ethanol concentration at constant 2 h extraction and 45 °C.

3D response surface curves can be used to provide a better understanding of interaction between any two factors. Response surface plots are generally the graphical representation of the regression equation. In the present study, each response surface presents the effect of any two factors on the wear loss while other two factors are held at the middle level. Figure 5a shows the interaction effect of time and temperature on amentoflavone yield while pressure and ethanol concentration are kept constant at values of 20 MPa and 80% (ethanol: water, v/v), respectively. This response surface plot indicates that longer extraction times and higher temperatures favor amentoflavone extraction. The maximum yield (4.48 mg/g) was obtained at extraction times longer than 2.35 h and temperatures above 48 °C. A longer extraction time was helpful for exhaustive extraction. Temperature can affect the extraction. The CO_2_ solvation power depends on solvent density, which decreases with temperature and increases with pressure. The phenomenon of increase in the yield with raising extraction temperature could be due to the enhancement in the solute (amentoflavone) vapor pressure with higher temperature, which was more significant than the reduction in the solvent density, increasing consequently the extraction temperature [13]. Similar effects of time and temperature on quercetin and ginkgetin yield can also be observed in Figure 6a and Figure 7a, respectively. Higher quercetin and ginkgetin yields occurred when the extraction time was longer and the extraction was performed at higher temperature. However, the interaction between time and temperature was not statistically significant (*p* > 0.05).

The effect of different combinations of extraction time and pressure on amentoflavone, quercetin and ginkgetin yield is shown in Figure 5b, Figure 6b and Figure 7b, respectively, while temperature and ethanol concentration are kept constant at values of 45 °C and 80% (v/v), respectively. It was observed from Figure 5b that higher yields (higher than 4.5 mg/g, 2.3 mg/g and 3.3 mg/g for amentoflavone, quercetin and ginkgetin, respectively) were attained by setting the extraction time at longer than 2 h and the pressure higher than 22 MPa. 

The pressure increase, at constant temperature, enhances the extraction yield due to the increased CO_2_ density and consequently in the solvating power. The interaction between extraction time and pressure was significant (*p* < 0.05) for amentoflavone extraction, while the interaction was not significant for quercetin and ginkgetin extraction.

Figure 5c, Figure 6c and Figure 7c illustrate the effect of extraction time and ethanol concentration on amentoflavone, quercetin and ginkgetin yield. The yield of amentoflavone was obviously affected by ethanol concentration. It was not suitable to use lower or higher concentration of ethanol for extraction of amentoflavone. The change of extraction yield with ethanol concentration could be explained by the fact of a similar polar solvent dissolving a similar polar solute. Higher yields can be attained when the polarity of the fluid matches the polarity of the compound to be extracted. Higher yields (>4.4%) of amentoflavone were obtained when thee ethanol concentration was in the 75% to 84% range and the extraction time was from 2.1 h to 2.5 h. Similar effects of ethanol concentration on quercetin and ginkgetin yield were observed. Higher yield (3.5 mg/g or 3.2 mg/g) was produced when the ethanol concentration was in the 74% to 83% range or in the 77% to 84% range for quercetin and ginkgetin, respectively. The interaction between time and ethanol concentration was statistically significant for ginkgetin extraction.

When considering the effects of temperature and pressure on the yields of amentoflavone, quercetin and ginkgetin, higher yields were attained at higher temperature and higher pressure as shown in Figure 5d, Figure 6d and Figure 7d. Higher yields occurred in the 45 to 50 °C temperature range and a pressure of 20 to 25 MPa for the extraction of the three components. The yield improvement could be the result of rising vapor pressure at higher temperature and increasing fluid density with elevated pressure [14]. The interaction between temperature and pressure was statistically significant for ginkgetin extraction.

Response surface plots in Figure 5e, Figure 6e and Figure 7e show the effects of temperature and ethanol concentration on the yields of amentoflavone, quercetin and ginkgetin, respectively. It was observed that the amount of amentoflavone, quercetin and ginkgetin extracted could be influenced by ethanol concentration. When the ethanol concentration was increased from 45% to 81%, the improvement of yields was from 4 to 4.3 mg/g. As ethanol concentration increased further, amentoflavone yield decreased while extraction time and pressure were kept at the middle level, as displayed in Figure 5e. Higher yields (>4.3 mg/g) were obtained by setting temperature higher than 42 °C and ethanol concentration between 76% and 84%. Similar effects of temperature and ethanol concentration on yields were observed for quercetin and ginkgetin.

The effects of pressure and ethanol concentration on the yields of amentoflavone, quercetin and ginkgetin are shown in Figure 5f, Figure 6f and Figure 7f, respectively. It was observed from Figure 4f that higher yields (higher than 4.3 mg/g) for amentoflavone were attained when the extraction pressure was set higher than 19 MPa and the ethanol concentration was between 74% and 85%. Similar conditions could also be used for the extraction of quercetin and ginkgetin (Figure 6f and Figure 7f, respectively). Pressure and ethanol concentration have a significant interaction for the extraction of the three components (*p* < 0.05).

Table 3 shows that the suitability of the predictive value derived from the estimated models under the optimal extraction conditions (time 2 h, temperature 48 °C, pressure 25 Mpa, ethanol concentration 78%, flow-rate 2 L/min CO_2_) and experimental values for amentoflavone, quercetin and ginkgetin yields. Experimental values were not significantly different from the predicted values within the 95% confidence interval. Amentoflavone, quercetin and ginkgetin purity in the extracts is also compared in Table 3. The purity obtained with ethanol-modified supercritical CO_2_ was four times higher than that with Soxhlet extraction. Furthermore, the yield obtained with ethanol-modified supercritical CO_2_ was higher than that obtained with Soxhlet extraction. This reveals that an exhaustive extraction was obtained with ethanol as co-solvent in supercritical CO_2_ extraction under the selected operation conditions.

**Table 3:** Extraction yields and purity of amentoflavone, quercetin and ginkgetin from the leaves of *T. chinensis*.

Extraction Condition	Amentoflavone		Quercetin		Ginkgetin	
Dynamic extraction time, 2 h; temperature, 48 °C; pressure, 25 Mpa; Ethanol concentration,78%; flow-rate, 2 L/min CO_2_	Yields (mg/g) (mean ± SD, n = 6)					
	Experimental	4.47 ± 0.06		3.65 ± 0.04		3.39 ± 0.02
	predicted	4.47		3.69		3.40
	Purity (%) (mean ± SD, n = 6)					
	9.41 ± 0.08		7.39 ± 0.05		6.02 ± 0.06	
Soxhlet extractor; extraction solvent, methanol; extraction time, 7 h	Yields (mg/g) (mean ± SD, n = 6)					
	4.08 ± 0.03		2.96 ± 0.03		2.17 ± 0.02	
	Purity (%) (mean ± SD, n = 6)					
	2.31 ± 0.02		1.83 ± 0.02		1.48 ± 0.02	

## 3. Experimental Section

### 3.1. Materials and Reagents

Leaves of *T. chinensis* were collected from five trees located at Ningbo Taxus Bio-engineering (29°18''N/117°32'E, Ningbo, China) on 15–20 September 2012. The leaves were ground into powder using a herbal pulverizer (FW 100, Tianjin Taisite Instrument Co. Ltd, Tianjia, China) and sieved through a 250 μm filter for extraction later. Quercetin, amentoflavone and ginkgetin standards were purchased from the National Institutes for Food and Drug Control (Beijing, China). CO_2_ (purity 99.5%) was supplied by Fangxin Gas Ltd. (Ningbo, China). Acetonitrile of HPLC grade was from Tedia Chemicals (Charlotte, NC, USA). Ethanol and methanol (analytical grade) were purchased from Sinopharm Chemical Reagent Co. Ltd. (Shanghai, China). Celite (chemical grade) was from Fengcheng Chemical Ltd. (Shanghai, China).

### 3.2. Supercritical Fluid Extraction

A supercritical fluid extractor Spe-ed SFE-2 (Applied Separation, Franklin, PA, USA) was used, which operates with two pumps, a master pump for delivery of CO_2_ and a second pump (Knauer pump, model K-501, Berlin, Germany) for the addition of co-solvent. An accurately weighed quantity of grounded sample (about 1 g) was placed in a 10 mL extraction vessel (60 × 15 mm, i.d.) and the void volume was filled with Celite. Before the extraction was started, the extraction vessel was preheated in the oven for 10 min. The extraction conditions were as follows: extraction time, static extraction for 5 min and then dynamic extraction up to 3 h; temperature, from 35 to 55 °C; pressure, from 10 to 30 MPa; Modifier ethanol concentration, 60% to 100% (ethanol/water, v/v); flow-rate of carbon dioxide (gaseous state), 2 L/min; flow-rate of modifier, 0.4 mL/min. Collection is at room temperature and atmospheric pressure. The extracts are collected in glass vials (30 mL containing 4 mL of methanol) with a rubber plug at the top. A metal extension to the metering valve is used to pierce the rubber plug and allow collection directly in the collection solvent. A hypodermic needle is pierced through the plug and connected to a flow meter. The extracts were quantitatively transferred to a 25 mL volumetric flask and made up to the mark with methanol. This solution was analyzed with UPLC.

### 3.3. Soxhlet Extraction

A known quantity of ground sample (2.0 g) was accurately weighed into a thimble and was extracted in a 50 mL extractor with 50 mL of methanol at a syphon rate of 1 cycle/ 15 min. After 7 h of extraction with the solvent, the extraction solvent was essentially colorless and the extracts were transferred to a 50 mL volumetric flask and made up to the mark with methanol. This solution was analyzed. All extracts were filtered through a 0.22 μm membrane filter before injecting into the UPLC system.

### 3.4. UPLC Analysis

Chromatography was performed using a UPLC system module 1290 infinity (Agilent, Santa Clara, CA, USA) equipped with photodiode array detector. ZORBAX Eclipse C18 column (2.1 × 150 mm, 1.8 μm particle size, Santa Clara, CA, USA) was used for separation and the column temperature was maintained at 30 °C. Detection was at a wavelength of 360 nm. Elution with solvent A (water) and solvent B (100% acetonitrile) in a step gradient manner at a flow rate of 0.3 mL/min was carried out as follows: 0–1 min, 10% B; 1–3 min, 10%–20% B; 3–6 min, 20%–45% B; 6–8 min, 45%–75% B; 8–15 min, 75% B; the sample injection volume was 1 μL. A series of standards (n = 6) in the range of 20–200 μg/mL, 63–630 μg/mL and 22–110 μg/mL for amentoflavone, quercetin and ginkgetin, respectively were prepared in methanol, giving linear calibration curves y = 1.4317x − 49.013, R^2^ = 0.9967; y = 1.7822x − 4.2832, R^2^ = 0.9989 and y = 1.1579x − 13.09, R^2^ = 0.9989 for them, respectively. The extraction yields of amentoflavone, quercetin and ginkgetin were calculated according to the calibration curves, respectively.

### 3.5. Experimental Design

Amentoflavone, quercetin and ginkgetin are classified as flavonoids, which are polar compounds. It is necessary to add small amounts of modifiers for extraction of the polar components in supercritical CO_2_ extraction [15]. In this work, four factors, *i.e.*, extraction time, temperature, pressure and different concentration of ethanol were studied for their effects on the extraction yield of the three components. A central composite design (CCD) with four variables at five levels was used, which allows the modeling of curvature within the factor space and improving the quality of prediction. Based on the CCD, thirty runs with six replicate in center points were considered [16]. The range of independent variables and their levels is presented in Table 4, which was based on the results of preliminary experiments. The software Design Expert (Stat-Ease Inc., Minneapolis, MN, USA) was employed for experimental design, data analysis, and model building. The matrix for the optimization experiment is summarized in Table 1. In this study, the experimental run was randomized in order to reduce the error arising from the experimental process.

The relationship between the response and the independent variables was calculated by the second-order polynomial equation (Equation (4)). The non-linear computer-generated quadratic model is used for this model:

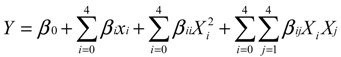
(4)
where Y is the predicted response; X_i_ and X_j_ are independent variables which influence the response variable Y; β_0_ is the offset term; β_i_ is the ith linear coefficient; β_ii_ is the ith quadratic coefficient; and β_ij_ is the ijth interaction coefficient.

**Table 4:** Factors and levels studied using CCD.

Factors	Coded Symbols	Levels				
		−2	−1	0	1	2
Extraction time (h)	X_1_	1	1.5	2	2.5	3
Extraction temperature (°C)	X_2_	35	40	45	50	55
Extraction pressure (MPa)	X_3_	10	15	20	25	30
Ethanol concentration (ethanol/water, v/v, %)	X_4_	60	70	80	90	100

## 4. Conclusions

The process parameters of SFE of amentoflavone, quercetin and ginkgetin from the leaves of *T. chinensis* were successfully optimized by response surface methodology. The optimum parameters for extraction of amentoflavone were 48 °C, 25 MPa, 2.02 h and 78.5% ethanol; 46 °C, 24 MPa, 2.3 h, 82% ethanol and 48 °C, 20 MPa, 2.38 h, 82% ethanol for the extraction of quercetin and ginkgetin, respectively. The maximum yield (4.47 mg/g) at optimized conditions for amentoflavone extraction (48 °C, 25 MPa, 2 h and 78% ethanol) could be obtained with higher yield for extraction of quercetin (3.65 mg/g) and ginkgetin (3.39 mg/g), respectively. The optimized parameters for the extraction of the three components are helpful for applications of the plant resource using the environmentally friendly SFE technique. The developed SFE method with UPLC analysis offers a suitable method of quality evaluation for the plant.
